# Cancer survivors’ needs during various treatment phases after multimodal treatment for colon cancer - is there a role for eHealth?

**DOI:** 10.1186/s12885-018-5105-z

**Published:** 2018-12-04

**Authors:** C. M. den Bakker, F. G. Schaafsma, J. A. F. Huirne, E. C. J. Consten, H. B. A. C. Stockmann, C. J. Rodenburg, G. J. de Klerk, H. J. Bonjer, J. R. Anema

**Affiliations:** 1 Department of Occupational and Public Health, VU University Medical Center, Amsterdam Public health institute, Van der Boechorststraat 7, 1081 BT Amsterdam, The Netherlands; 2 Department of Surgery, VU University Medical Center, Amsterdam, The Netherlands; 3 Department of Gynecology, VU University Medical Center, Amsterdam, The Netherlands; 4 Department of Surgery, Meander Medical Center, Amersfoort, The Netherlands; 5 Department of Surgery, Spaarne Gasthuis, Hoofddorp, The Netherlands; 6 Department of Medical Oncology, Meander Medical Center, Amersfoort, The Netherlands; 7 Department of Medical Oncology, Spaarne Gasthuis, Hoofddorp, The Netherlands

**Keywords:** Colon cancer, Multimodal treatment, Recovery, Patient reported outcomes, Cancer care. eHealth

## Abstract

**Background:**

More colon cancer patients are expected to fully recover after treatment due to earlier detection of cancer and improvements in general health- and cancer care. The objective of this study was to gather participants’ experiences with full recovery in the different treatment phases of multimodal treatment and to identify their needs during these phases. The second aim was to propose and evaluate possible solutions for unmet needs by the introduction of eHealth.

**Methods:**

A qualitative study based on two focus group discussions with 22 participants was performed. The validated Supportive Care Needs Survey and the Cancer Treatment Survey were used to form the topic list. The verbatim transcripts were analyzed with Atlas.ti. 7th version comprising open, axial and selective coding. The guidelines of the consolidated criteria for reporting qualitative research (COREQ) were used.

**Results:**

Experiences with the treatment for colon cancer were in general positive. Most important unmet needs were ‘*receiving information about the total duration of side effects’, ‘receiving information about the minimum amount of chemo needed to overall survival’* and *‘receiving a longer aftercare period* *(with additional attention for psychological guidance)’*. More provision of information online, a chat function with the oncological nurse specialist via a website, and access to scientific articles regarding the optimal dose of chemotherapy were often mentioned as worthwhile additions to the current health care for colon cancer.

**Conclusions:**

Many of the unmet needs of colon cancer survivors occur during the adjuvant treatment phase and thereafter. To further optimize recovery and cancer care, it is necessary to have more focus on these unmet needs. More attention for identifying patients’ problems and side-effects during chemotherapy; and identifying patients’ supportive care needs after finishing chemotherapy are necessary. For some of these needs, eHealth in the form of blended care will be a possible solution.

## Background

More colon cancer patients are expected to fully recover as a result of improvement of general healthcare, optimization of cancer treatment and increased earlier detection of colorectal cancer through screening programs [1–6]. Attempts to improve postoperative recovery tended to be focused on the intraoperative period (e.g. laparoscopic surgery) and the immediate postoperative period (e.g. enhanced recovery pathways) [7–9]. The required time for full postoperative recovery after colon cancer surgery is not yet determined [10, 11]. Colon surgery is complemented with adjuvant chemotherapy (multimodal treatment) for 30–40% of patients. Multimodal treatment (from making the diagnosis at the first presentation at the outpatient clinic until the last cycle of chemotherapy) can take up to 12 months in total, resulting in a more extended period before full recovery is reached [12].

There are also negative developments of general health- and cancer care such as decreased availability of healthcare providers resulting in less availability for patients at the outpatient clinic, but also centralization of care increasing the distance to hospitals and increasing overall health care costs [13–16]. Care of patients with chronic diseases is fragmented resulting in among others incomplete delivery of information and in less support from health care professionals how to deal with problems [17]. This development also applies to colon cancer care as a strain on health service provision is placed due to increasing survival rates resulting in similar needs and problems regarding care (e.g. guidance, communication and monitoring) [18–20]. The care of cancer survivors is transferred to the community and survivors will be encouraged to play an active role in their own care themselves [21].

eHealth can be helpful to solve some of the unmet needs in general health- and cancer care [22, 23]. To illustrate, health information can continuously be accessed online via eHealth tools, and these can also be used for interactive communication purposes. eHealth provides opportunities for self-management and helps optimizing the continuity of care by improving long-term monitoring [24]. By empowering patients, they will achieve an active role in managing their own care [25, 26]. Benefits of eHealth solutions in cancer care include improved well-being, better patient-clinician communication, and lower symptom distress [27–29].

To date, no studies among colon cancer patients have focused on full return to normal activities or societal participation after multimodal treatment. Endpoints of colon cancer surgery are currently often defined in terms of short-term outcomes (e.g. in-hospital stay or morbidity) or overall mortality whereas long-term patient reported outcomes are becoming increasingly important [2, 7–11]. In addition, literature about the experiences of colon cancer patients who have received multimodal treatment is insufficient [19–21]. The first aim of this qualitative study was to gather participants’ experiences with their full recovery in the different treatment phases and identifying their needs experienced during these phases. The second aim was to explore possible solutions for any unmet needs by the introduction of eHealth.

## Methods

### Study design and setting

This study has a qualitative research design based on focus group discussions (FGDs). This method is suitable for investigating experiences, attitudes and emerging ideas from a group [30]. In this study, FGDs were used to have an interactive discussion exploring participants’ experiences with recovery and identifying needs of the entire colon cancer treatment period during the different treatment phases. In addition, FGDs make it possible to discuss solutions, e.g. by the introduction of eHealth, for the identified unmet needs. The treatment phases were divided in the i. perioperative phase (receiving the diagnosis, preparing for and having surgery); ii. during chemotherapy phase (surgery complemented with adjuvant chemotherapy); iii. After chemotherapy phase (follow up period with visits at an outpatient clinic). This design with only participants who had received multimodal treatment enabled the researchers to discuss the cancer care provided by multiple health care professionals more extensively. The guidelines of the consolidated criteria for reporting qualitative research (COREQ) were used [31].

### Study participants

Purposeful sampling was used to select the study participants. Patients aged over 18 years with a diagnosis of colon cancer who had undergone colon surgery between 2014 and 2016, and who had finished complementary chemotherapy (multimodal treatment) were eligible. These patients were all cancer survivors. Patients needed to master the Dutch language fluently. Patients receiving neoadjuvant chemo radiation were not of interest because this is another subpopulation with probably a different recovery trajectory.

### Recruitment strategy

Participants were recruited by the oncological nurse specialist from the patient files of the Meander Medical Center Amersfoort and Spaarne Gasthuis Haarlem & Hoofddorp. Both institutions are seen as top clinical hospitals in the Netherlands, and adequately represent the colon care as is given in the Netherlands. Colon care in the Netherlands is organized as follows. Via the general practitioner or the national screening program patients will be referred to the gastroenterologist for a colonoscopy. The results of the colonoscopy will be discussed with a surgeon, and patients will also be guided in this process by a colorectal nurse specialist. After surgery, in case of complementary therapy, treatment guidance will be continued by an oncological nurse specialist. The chemotherapy will be coordinated by an oncologist. After finishing chemotherapy, patients’ follow up schedule up to five years will be coordinated by a surgeon.

Oral informed consent was obtained by telephone and written informed consent including permission for audio recordings and a disclosure agreement was obtained before the start of the FGD.

### Data collection

The medical ethics committee of the VU University medical center Amsterdam approved the protocol of this study in 2015 (registration number 2014.301). In the preparation phase for the FGDs, overarching themes were created based on literature and discussion with the project team. The Supportive Care Needs Survey (SCNS) and the Cancer Treatment Survey (CaTS) were used in this preparation phase to optimize the topic list [32, 33]. The SCNS measures the need and level of need of supportive care during treatment of cancer patients. The CaTS assesses the adequacy of preparation for cancer treatment, specifically chemotherapy and radiotherapy. By combining these two surveys a topic list was created and it was expected to obtain the most practical and complete information from participants. After identifying participants’ (un)met needs, possible solutions for these needs were explored by introducing several eHealth solutions during the second part of each FGD. The predefined discussion topics during the FGD were supplied by the researcher (FS) who acts as ‘moderator’ during the discussions. Under these conditions, the moderator undertakes a guiding role in facilitating discussion rather than simply interviewing.

### Data analysis

The two FGDs were audio taped, transcribed verbatim and coded with Atlas.ti 7. Each participant was allocated a study number, all names were removed from the transcripts to ensure an anonymous analysis. The transcribed interviews were reviewed and coded independently by two researchers (CdB and FS) of the project team. The verbatim transcripts were analyzed comprising open, axial and selective coding as described in the grounded theory approach [34]. After review of the transcripts and before coding started, codes were discussed and further modified by CdB and FS until consensus about the code guide book was reached. Firstly, CdB and FS both coded the transcripts with open coding, including in-depth coding, based on the topic guide book. Hereafter, CdB and FS discussed the results in two sessions to create subthemes by relating codes to each other resulted in axial coding of the data. In a separate session with HA these axial coding were evaluated and discussed in order to develop a storyline by relating subthemes to the main themes. This resulted in selective coding. Furthermore, the level of data saturation was systematically studied. The frequency of the quotes within each theme and their distribution across the focus groups was explored, based on a data saturation approach as described by Guest et al. [35]. In addition, the themes were informally evaluated by the researchers and moderator to discuss if new results had been reported during the second focus group. Cited quotes were translated directly from Dutch and were added to illustrate the themes. Data about demographics and disease/treatment of participants were analyzed by using SPSS inc., Chicago, IL, USA, v22.0.

## Results

### Participants characteristics

In total, 75 patients (37 in Meander Medical Center and 38 in Spaarne Gasthuis) were eligible for participating in the study and received an information letter. Thirty patients were willing to participate (response rate of 40%) of which 22 were available to participate on the scheduled dates of the FGDs. Demographic- and disease- / treatment related characteristics of the participants of both focus group discussion are presented in Table 1. After two FGDs, an equal distribution between men and women was reached. The median age was 65.3 years (34.9–76.2). Median time after surgery was 14.9 months (8.3–22.1) and median time after finishing the last course of chemotherapy was 8.7 months (2.3–17.22). Sixteen participants had laparoscopic surgery, four had an intended open approach and two a perioperative conversion. All patients had a stage III tumour. Four participants received a stoma during surgery. More than half of the participants had a comorbidity and five participants had minor postoperative complications. A combination of Capecitabine and Oxaliplatin was the chemotherapy regimen of choice of all included participants.

**Table 1:** Baseline characteristics

Study number	Gender	Age at operation (ranges in years)	Comorbiditiy	Operation	Postoperative complications	Age at focus group (years)	Time after operation (months)	Time after last course of CTx (months)	8 courses of CTx received	Stoma
FG1-R1	Male	65–70	Yes	Laparascopic	No	65–70	10	5	No	No
FG1-R2	Female	40–45	No	Open procedure	No	40–45	14	6	Yes, with adjusted dose	Yes
FG1-R3	Male	NA	No	Conversion to open	Yes	75–80	NA	4	Yes, with adjusted dose	No
FG1-R4	Female	60–65	Yes	Laparascopic	No	60–65	17	11	Yes, with adjusted dose	No
FG1-R5	Female	60–65	Yes	Laparascopic	No	60–65	17	10	Yes, with adjusted dose	No
FG1-R6	Male	NA	No	Laparascopic	No	70–75	NA	11	Yes, with adjusted dose	No
FG1-R7	Male	65–70	Yes	Laparascopic	No	65–70	16	11	No	Yes
FG1-R8	Male	60–65	No	Laparascopic	No	60–65	16	12	Yes, with adjusted dose	No
FG1-R9	Male	70–75	Yes	Open procedure	Yes	70–75	12	4	Yes, with adjusted dose	No
FG1-R10	Male	60–65	Yes	Laparascopic	No	60–65	11	4	Yes	No
FG1-R11	Female	30–35	No	Conversion to open	Yes	30–35	22	15	Yes, with adjusted dose	No
FG2-R1	Female	75–80	Yes	Laparascopic	No	75–80	8	2	Yes	No
FG2-R2	Female	50–55	Yes	Laparascopic	No	55–60	14	10	No	No
FG2-R3	Male	65–70	No	Laparascopic	Yes	65–70	16	9	Yes	No
FG2-R4	Male	50–55	Yes	Laparascopic	No	50–55	14	8	Yes, with adjusted dose	No
FG2-R5	Male	55–60	Yes	Open procedure	No	55–60	11	4	No	Yes
FG2-R6	Female	55–60	No	Laparascopic	No	55–60	12	6	Yes, with adjusted dose	No
FG2-R7	Female	65–70	Yes	Laparascopic	No	65–70	20	12	No	No
FG2-R8	Female	65–70	Yes	Laparascopic	No	65–70	22	15	Yes, with adjusted dose	No
FG2-R9	Male	60–65	Yes	Laparascopic	No	65–60	20	17	No	No
FG2-R10	Female	55–60	No	Laparascopic	No	55–60	15	9	Yes, with adjusted dose	No
FG2-R11	Female	70–75	No	Open procedure	Yes	70–75	15	8	Yes	Yes

*FG1* Focus group discussion 1, *FG2* Focus group discussion 2, *R* Respondent number

### Exploring recovery paths and identifying (un)met needs

#### Perioperative phase

##### Recovery path

**Fig. 1:**
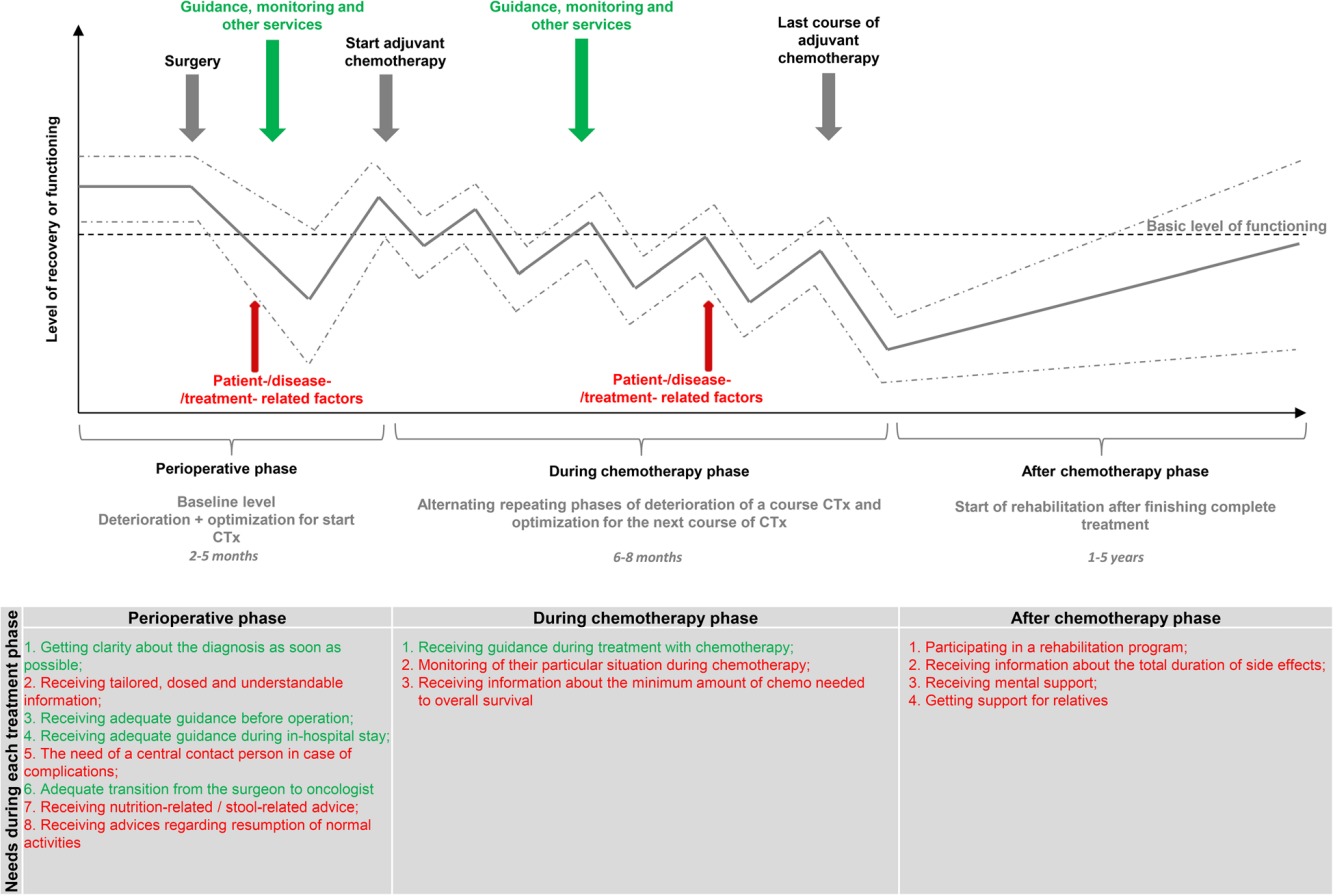
Proposed recovery paths according to patients after multimodal treatment for colon cancer including all needs per treatment phase. Met needs are presented in green, unmet needs in red. CTx = Chemotherapy

In cases without complications a quick physical recovery after hospital discharge was reported by the majority of the participants (see Fig. 1). None of the participants indicated emotional complaints during this period. The postoperative phase was experienced as a relatively short recovery period, even in case of an open surgical approach or in case of complications or comorbidities (see Fig. 1). Participants reported more discomfort of the colon cancer diagnosis itself than the surgical procedure that followed. Some participants were able to exercise in this postoperative phase to prepare for the chemotherapy.

##### Needs assessment of perioperative phase

*Getting clarity about the diagnosis as soon as possible - met need.*

When a participant was referred to the hospital via the general practitioner or the colon cancer screening program, participants rated it important to receive clarity about the diagnosis as soon as possible. Most participants experienced the time between referral to the hospital and the diagnosis as relatively short. They indicated that this resulted in rapid transparency and less uncertainty about their situation. This enabled them to start preparing for the treatment phase.

*Receiving tailored, dosed and understandable information - unmet need.*

Participants need information about the colon cancer diagnosis, operation and possible complications in a tailored, dosed and understandable form. In many cases, the cancer location was drawn on paper by the surgeon which created a lot of clarity for many participants about the surgical procedure. Information about possible complications and the risk of receiving a permanent or temporarily stoma ensured reassurance in most cases. However, the amount of information about diagnosis and treatment was often experienced as too extensive. Most participants indicated that the shock inflicted by receiving the diagnosis prevented absorbing detailed information. In addition, some participants reported that the information was too difficult to process. They would appreciate more tailored and personalized information to their needs and about their specific diagnosis; and that the amount of information should be temporized an dosed.

*“There is only one thing that matters if you just hear that diagnosis, it’s like receiving a slap in the face. If the doctor then tells you all that information, you no longer hear it. Because you're so busy with yourself and the cancer diagnosis, fortunately my wife was sitting next to me” (FG1-R7)*

*Receiving adequate guidance before operation – met need.*

Adequate guidance in the perioperative phase was needed for an optimal preparation for the surgical procedure. If participants had any questions about the operation, they preferred to have a single contact person. The colorectal nurse specialist was rated as an important link in optimal preparation for surgery. The participants indicated that this need was met resulting in, among others, more confidence about the operation.

*Receiving adequate guidance during in-hospital stay – met need.*

Adequate guidance during the in-hospital stay was needed for an optimal preparation for discharge and recovery at home. The guidance during admission was experienced as very positive. After surgery, the surgeon provided additional information about the procedure resulting in clarity and reassurance. Participants felt very supported by the colorectal nurse specialist as point of contact in case of questions or additional problems. If participants were afraid to go home, the surgeon allowed participants to stay longer in the hospital creating a safe feeling. During admission, enough attention was paid to the participants’ bowel habits and diet. The guidance of a physiotherapist during the first days after surgery was experienced as very pleasant.

*The need of a central contact person in case of complications – unmet need.*

When participants suffered from surgical complications, they liked to see a central contact person who takes the lead throughout the process. When they had to visit different health care professionals for each specific complication it felt that they had to take control of their treatment themselves. They had to mention their complications to each different health care professional in order to get the right treatment at the right moment resulting in an unsafe feeling for participants with postoperative complications.

*“What I missed in the period after surgery and before the start of chemotherapy is actually still a kind of central control over what was happening to me. The urologist is mainly busy with the bladder, the oncologist who wants start chemotherapy but cannot start it yet due to the complications with the bladder and the surgeon has to do many other things. So I felt that I had to really take care of myself and what happened to me and that I had to intervene myself not knowing whether it was necessary or not.” (FG1-R3)*

*Adequate transition from the surgeon to oncologist – met need*

At time of discharge, participants were told that their specific case would be discussed postoperatively in a multidisciplinary meeting with involvement of all treating health care professionals. Knowing that the oncologist was well informed about their case improved participants’ confidence in the coordination of care before starting adjuvant chemotherapy.

*Receiving nutrition-related / stool-related advice – unmet need*

Participants needed information that a surgical procedure may have a long-term impact on bowel function. Furthermore, dietary advice for the period after surgical resection seemed very important for participants. Some participants received guidance for a limited period from a dietician. However, this was mainly to improve recuperation postoperative. Others received limited to no dietary advices when leaving the hospital. Participants perceived a strong link between dietary intake and bowel function and they would like to have more advice and guidance on this topic. They do not know who to consult and therefore, unfortunately, participants often reported uncertainty related to their diet and bowel function.

*“After surgery I had a dietician, she said you can basically just eat everything but limited processed meat or nothing at all. Which was clear to me. But I still have to go very often to the toilet. Often to defecate. Sometimes it’s just that urge to defecate and then nothing happens. And then you'll go back thinking has that to do again with food? I try to keep it to myself, and see if it happens with specific food more often. Well then I see some pattern in it. At one point I felt a little bit alone with these problems.” (FG1-R11)*

*Receiving advices regarding resumption of normal activities – unmet need.*

Participants indicated that clear advice regarding resumption of normal activities was needed to resume daily activities. Participants perceived the information received as too limited or inconsistent. Some participants did not perform certain daily activities due to uncertainty. Others take the comment ‘You can do everything again’ too literally resulting in unnecessary discomfort.

*“I went grocery shopping by foot, using a bag, which I expected that I could do. However this clearly was not yet possible, so I had to ask several times to a bystander if they could help. If someone had told me when I would have been able to do this after surgery I would have known what to expect.” (FG2-R2)*

#### During chemotherapy phase

##### Recovery path

Participants described that they needed a few days of recovery time after a course of chemotherapy, but that they handled that well (see Fig. 1). However, after every additional course of chemotherapy the required period of recovery lasted longer. After course 5 or 6 the recovery process became more severe, more side-effects were reported and the duration of recovery lasted even longer (see Fig. 1). In addition, each participant started reporting severe long-lasting side-effects of the chemotherapy. As a result, for the majority of participants the dose of chemotherapy was reduced or the chemotherapy stopped prematurely.

##### Needs assessment of during chemotherapy phase

*Receiving guidance during treatment with chemotherapy – met need.*

Before and during treatment with chemotherapy, participants need someone who can answer their questions about associated problems, uncertainties and side-effects. The oncological nurse specialist played an important role for many participants. Most participants had a consult by a nurse shortly after surgery at the outpatient clinic. During the first consultation, participants received both verbal and written information about the complementary treatment. In addition, they received phone numbers of the oncological nurse specialist they could call 24/7 with questions and problems. The opportunity to readily consult the oncological nurse specialist created much confidence.

*Monitoring of their particular situation during chemotherapy – unmet need.*

Participants needed more monitoring of side-effects during chemotherapy. Increasingly severe side-effects over the course of treatment resulted in an increased need for in-depth information. Participants reported that they often did not had an appointment when symptoms emerge. Moreover, if they had an appointment with their oncologist participants were hesitant to list complaints as it had become less acute or fearing it might be considered trivial. The level of monitoring during treatment did not match participants’ need. Participants reported a limited time available during an appointment with the oncologist or the oncological nurse specialist, whereas the participant often needed a longer conversation. This perceived neglect resulted in participants deciding to stop reporting symptoms.

*“The oncologist seems a bit too busy to me, because he starts asking questions in the waiting room and then you walk with him to his consulting room. And when you're in that room you can almost go home again, with him looking at his watch. He is always ahead of his schedule. And then you think you better can make a list with questions in advance, otherwise it will not be useful. The doctor is often talking to you towards the door.” (FG1-R1)*

*Receiving information about the minimum amount of chemo needed to overall survival – unmet need.*

Participants frequently discussed situations in which the dosage of a course of chemotherapy was adjusted downwards or stopped. They needed information and explanations about the reasons why chemotherapy was adjusted or stopped. The participants felt they received insufficient information and/or inadequate explanations. They found that they were not well enough informed about the consequences of a lower dose of chemotherapy. Participants also indicated that they wanted to have more involvement in this decision, but that they often felt unable to make this choice themselves which makes it difficult to facilitate their wish for shared decision making. This lack of guidance and involvement resulted in increased uncertainty.

*“You assume what the internist says is the best option and he explains the options and tells me that I do not have to say ‘yes’. ‘What do you think about it?’, Of course I say ‘yes’, you accept everything. You can’t say ‘no’. I think you have no choice.” (FG1-R7)*

#### After chemotherapy phase

##### Recovery path

After finishing the chemotherapy, participants physical and emotional functioning is at the lowest point and only gradually increasing (see Fig. 1). According to the participants, this period lasts for a long time. After finishing chemotherapy, a significant number of complaints were reported. Most frequent was neuropathy in hands and feet adversely affecting the ability to perform everyday activities. In addition, participants frequently reported diminished brain functions including short-term memory problems and obliviousness. Participants related these complaints to the number of chemotherapy treatments. During this period emotional complaints are both more frequent and prominent. All participants indicated that the recovery process was ongoing at the time of the FGD (between 2 months and 2 years after finishing the chemotherapy treatment). During the FGDs participants suggested that the duration and severity of this recovery period can probably be influenced by patient- (e.g. age), disease- (e.g. comorbidities) and treatment related factors (e.g. severity of side-effects).

##### Needs assessment of after chemotherapy phase

*Receiving a longer aftercare period – unmet need*

After the last course of chemotherapy, a rehabilitation program was needed. This rehabilitation program was not offered to all participants. These rehabilitation programs are rated very positively by the participants of the study who participated in these programs. Being able to interact with patients with similar cancer-related problems in group sessions but also physical therapy and access to a social worker were rated highly by these participants. However, participants felt an acute stop of support at the moment the program ended whereas their needs did not end at that point in time. They preferred to participate in rehabilitation programs till more than a year after finishing chemotherapy.

*“But I miss the aftercare. Occasionally I think I'd like to take that phone and just like during the process where I could talk very well with the oncology nurse specialist. What would I still like to have feedback from her again. Then you lose the negative tension and then you'll be able to resist it again.” (FG1-R4)*

*Receiving information about the total duration of side effects – unmet need.*

A need for information about total duration of side effects was mentioned by all participants. No or limited information was given about the potential duration of side-effects after chemotherapy. Most participants were still not able to perform all their daily activities, even after more than one year after the last course. This is something participants find difficult to accept. The difficulties resuming life resulted in a lower quality of life. In retrospect participants reported that they would have preferred less courses of chemotherapy if they would have known the duration upfront. Participants also reported frequently they felt unsecure as a result of a perceived lack of adequate advice on this topic by health care professionals.

*“And I feel like I'm beginning now and that I start to find some kind of balance between accepting that my life will never be the same as 2 years ago and that things have deteriorated. However I'm still building a valuable life again.” (FG1-R8)*

*Receiving emotional support – unmet need*

In this treatment phase, participants reported a need for emotional support because these complaints became more dominant. Although participants’ periodic visits are well arranged in the follow-up period postoperatively, these visits are mainly focused on the progress of health recovery and fail to address emotional problems or complaints of participants. Participants are often only able to find emotional closure at the end of the entire treatment period. Participants would like to receive more emotional support from the health care professional or a referral to a specialist in this field, however this is omitted in the after-care participants receive.

*“The interesting thing is that we get medical examinations every six months 5 to 7 years long, but psychologically nothing is offered. While that's your biggest problem.” (FG1-R8)*

*Getting support for relatives – unmet need*

CRC diagnosis and treatment is completely focused on patients. However, the participants indicated that the impact on the relatives (can be both family and friends) is also severe and requires support. Relatives were only expected to support the participant but relatives also require support and guidance to cope with the consequences of the disease and treatment. Several participants reported emotional problems for relatives, even at stage where the participants were able to resume life as before the CRC diagnosis. They would like to see more attention for guiding relatives in this period.

*“I personally think that it is also good for family to have a conversation after or during chemotherapy, without the patient. To explain what is going on and what happens to your partner. During the conversation I was also anxious, it was uncomfortable. I think it is important that for the husband or wife or friend, there is also an opportunity that they can express themselves as well.” (FG1-R7)*

### Evaluation of unmet needs and problem solving by application of eHealth services

No participant had the same experiences with the recovery process / path as seen in Fig. 1. The ‘*during*’ and ‘*after chemotherapy phases*’ were seen as the most difficult stages of the recovery process according to participants.

Possible solutions for the reported unmet needs by providing eHealth services were reviewed by participants and provided in Table 2. Not all participants were immediately enthusiastic about using eHealth in their treatment process. Some participants were already familiar with eHealth and they were more open to the use of eHealth during the different treatment phases for colon cancer. The overall opinion of participants regarding eHealth services was that this should be supportive to and not a substitute of personal interaction with health care providers. There was a general preference by the participants for an online health care system that is 24/7 readily available for patients. Such an online health care system should also allow extended care and guidance even after closure of the chemotherapy courses. Since there is already a lot of information on the internet about colon cancer and its treatment, it was sometimes hard for participants to differentiate what information is correct. Recommendation of a specific eHealth tool by their own health care professional would enhance the perception of safety and therefore increase usage. After discussions between participants some more specific solutions for the identified unmet needs were provided (Table 2). These solutions were 1) more provision of information online that can be consulted 24/7 in a timeframe that fits the individual preference, and is tailored to the personal situation and needs, 2) a chat function with the oncological nurse specialist via a website, and 3) access to scientific articles regarding the optimal dose of chemotherapy were often mentioned solutions.

**Table 2:**
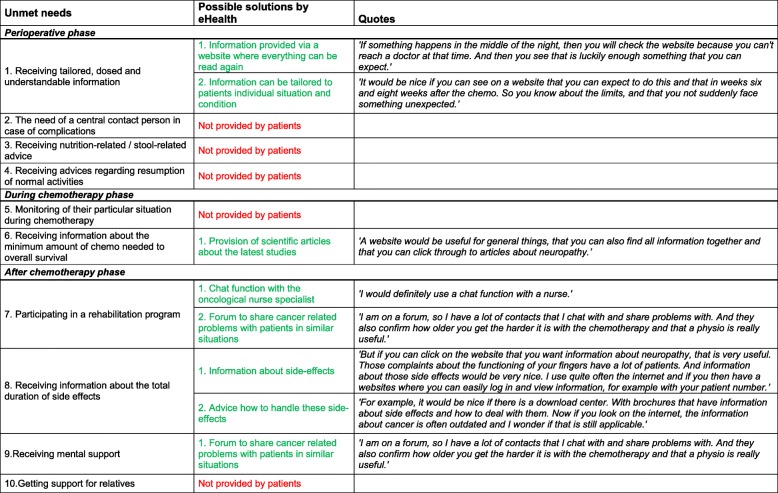
All mentioned unmet needs including possible solutions by eHealth & ICT according to the participants

Provided solutions are presented in green, in case no solution was mentioned these answers are presented in red

## Discussion

Colon cancer care in patients receiving surgery and chemotherapy can be divided in three main phases; the perioperative, the during and after finishing chemotherapy phases. The latter two phases were seen as most challenging phases, which is underlined by the high rate of unmet needs in this study. Most participants were satisfied with the guidance, communication and monitoring during their perioperative treatment phase. ‘*Receiving tailored, dosed and understandable information’* is an important unmet need in this phase. The most frequently mentioned unmet needs of the during and after chemotherapy treatment phases were ‘*receiving information about the minimum amount of chemo needed to overall survival’*, ‘*receiving information about the total duration of side effects’* and *‘receiving a longer aftercare period (with additional attention for psychological guidance)’*. More information online, a chat function with the oncological nurse specialist and access to scientific articles regarding the optimal dose of chemotherapy were often mentioned by participants as potential eHealth solutions for these unmet needs. Despite the potential eHealth solutions, participants frequently indicated that eHealth could only be a support but not a replacement of physical interaction as this remains very important.

Each recovery path is unique and it can be concluded that this, among others, depends on several patient- and disease- or treatment related factors. Despite these differences, the recovery path follows approximately the same direction as illustrated in Fig. 1. Most unmet needs were reported in the during and after chemotherapy phases which were evaluated as the longest and most severe periods of recovery. Recovery of patients is often discussed based on several theories regarding postoperative recovery [36–41]. Based on the results of this study it can be concluded that recovery for this specific patient population does not stop after surgery and also not after completion of the last course of chemotherapy. Full recovery for this population could be considered as handling the several survival phases described by Mullan et al. [42]. This theory is better equipped to evaluate a prolonged recovery period after multimodal treatment. The survival phases include an acute, an extended and a permanent survival phase. The first phase of this theory is the acute survival phase which is dominated by the cancer diagnosis and surgical treatment. According to the theory, and confirmed by our study results, this phase is described as the shortest phase. The extended phase is not predominantly a medical phase in which patients enter a phase of intermittent therapy (e.g. adjuvant chemotherapy) and periodic examinations. This phase is primarily psychologically dominated by the fear of recurrence of the cancer. This description fits with the participants of our study, as they were interviewed 2 months up to 2 years after finishing chemotherapy treatment and thus not yet arrived in the last (extended) survival phase. In this extended phase patients are considered survivors as the likelihood of recurrence is sufficiently small, and as a consequence can start resuming their lives completely.

Participants’ met needs, resulting in positive experiences with received care, could be attributed to the high quality of cancer care in the Netherlands. The nationwide Dutch Surgical Colorectal Audit (DSCA) was initiated in 2009 by the Association of Surgeons of the Netherlands to monitor, evaluate and improve colorectal cancer care which focusses on a set of process indicators, quality indicators and short-term postoperative outcomes [43, 44]. The quality of guideline compliance and clinical outcomes for colorectal cancer patients in the Netherlands improved significantly [44]. This is also reflected in the results of the *‘perioperative phase’* of this study where many met needs resulting in more positive experiences were reported compared to the later treatment phases. The mentioned unmet needs are in line with results of earlier published articles [18–20, 45–48]. ‘*Receiving tailored, dosed and understandable information’* is often mentioned in existing literature on patient expectations and preferences for information provision [46–48]. These studies also reported that patients’ preference was to receive information dosed during routine visits rather than hearing about long-term implications at the first consult whilst simultaneously trying to cope with their diagnosis [45]. Brown et al. emphasized the need of increased information provision during the entire treatment process [45]. In the review of Kortonoulas et al., ‘*receiving information about the minimum amount of chemo needed to overall survival’*, ‘*receiving information about the total duration of side effects’* and *‘receiving a longer aftercare period (with additional attention for psychological guidance)’* were also all ranked in the top ten of most prominent individual unmet needs [19].

Participants in other studies were often positive about an eHealth application that enables them to monitor their symptoms, provides advices and a tailored supportive care [49]. The main concerns raised were the potential effect on their privacy [50, 51]. Participants of our study preferred permanent face-to-face contact because they feared that an eHealth application would have an adverse impact on the relationship with health care professionals. Patients have more trust in physicians than in eHealth applications to provide relevant advice [52]. This can be attributed to the higher median age of our participants [53], and to the fact that the majority of these participants did not have any experiences with an eHealth program. Participants did see a possibility in the use of eHealth in cancer care in the form of blended care, in which the use of an eHealth application is additional to traditional care [49–53]. In line with literature, our participants reported that readily available information by eHealth could be a big advantage to solve the unmet need *‘receiving tailored, dosed and understandable information’* [49–53].

To our knowledge, this is the first qualitative study investigating full recovery during and after the entire treatment trajectory of colon cancer patients treated with multimodal treatment. One of the strengths of this study is that participants’ needs were investigated by FGDs with patients who had similar treatment trajectories. Previous assessments of patients’ needs were often administered through cross-sectional mailed surveys or via interviews with a variety of patient populations at different stages of the disease trajectory. FGDs are a more suitable way to obtain information by discussing different experiences of patients. Another strength of this study is the methodological quality, ensured by following the COREQ guidelines for reporting about qualitative studies [31]. In addition, to gather more information about patients’ experiences with supportive care and needs for optimal preparation for adjuvant therapy, the validated SCNS and CaTS were combined in the topics and questions for both FGDs. These surveys are widely used in investigating experiences of cancers patients [32, 33].

A possible limitation of this qualitative study is that, in general, more patients with outspoken negative or positive experiences participate in this kind of research than those with a more general view [54]. The reported results should therefore be caveated within this context. Generalization is not the objective of qualitative studies. However that the study population is a representative sample, the lack of wide racial, ethnic or cultural diversity within this study population, the lack of spread in socioeconomic status and the small subset of survivors limits the transferability of the results. Another limitation is that most participants in this study did not have much experience with eHealth, which may have affected the limited response to this topic within the FGD’s.

The results of our study provides health care professionals tools to optimize the care provided to patients with colon cancer undergoing surgery and chemotherapy. They could proactively ask more in-depth questions to discuss personal problems or treatment related side-effects in the *during* and *after chemotherapy phase*. This will help to identify patient personal needs in these treatment phases. The identified unmet need *‘monitoring of their particular situation during chemotherapy’* can be optimized by integration of Patient Reported Outcomes Measurements (PROMs) into the routine care of patients [55]. In the study of Basch et al. the PROMs were obtained via a web-based PROM platform [56]. By integrating the PROMs with the unmet needs related to more information about chemotherapy, overall survival and side effects may increase toleration to this treatment phase [56]*.*

Another benefit of asking patients more in-depth questions after finishing chemotherapy can be earlier detection of patients’ supportive care needs. If necessary, patients can then be referred to various cancer survivorship services that patients are often not aware of [46]. To answer to the wish to receive longer aftercare by patients, an eHealth self-management application developed to support cancer survivors in finding and obtaining optimal supportive care could be suggested [49, 57].

Quality indicators introduced by the DSCA audit have become more important in optimizing surgical cancer care. The positive impact on needs in the perioperative phase is confirmed in this study. Most important unmet needs were seen in the *during* and *after chemotherapy phase* at the moment there are no guidelines for quality indicators regarding chemotherapy treatment and related follow-up. There is a clear need to perform more research on the implementation of quality indicators for chemotherapy treatment. By standardizing and improving treatment facilitates including clear counseling and tailored information, the identified patients’ unmet needs during and after the chemotherapy phase can be reduced.

## Conclusion

Most frequently mentioned unmet needs are in the *during* and *after chemotherapy* phase and are correlated with an extended recovery path. To further optimize recovery and cancer care, it is necessary to have more focus on the unmet needs as described in this article. More attention for identifying patients’ problems and side-effects during chemotherapy; and identifying patients’ supportive care needs after finishing chemotherapy are necessary. According to participants, eHealth in the form of blended care can have a role to solve most of the identified unmet needs in the treatment of colon cancer.

## Abbreviations

CaTS: Cancer treatment survey
DSCA: Dutch surgical colorectal audit
FGDs: Focus group discussions
PROMs: Patient reported outcomes measurements
SCNS: Supportive care needs survey
